# Chaplaincy Perspectives on the Role of Spirituality in Australian Health and Aged Care

**DOI:** 10.1007/s10943-023-01752-4

**Published:** 2023-02-04

**Authors:** Megan C. Best, Kate Jones, Kate Bradford, Matthew Kearney

**Affiliations:** 1 Institute for Ethics and Society, University of Notre Dame Australia, Broadway NSW 2007, P.O. Box 944, Sydney, Australia; 2 Westmead Hospital, Sydney, Australia; 3 St Vincent’s Hospital, Sydney, Australia

**Keywords:** Chaplain, Healthcare, Aged care, Spirituality, Perspectives, Ricoeur, Metaphor, Narrative, Aboriginal

## Abstract

The aim of this study was to explore Australian chaplains’ views of spirituality. Semi-structured online interviews were conducted with 16 participants. Participants relied heavily on metaphors and analogies to describe spirituality. Four inter-related themes were identified through reflexive thematic analysis: (1) The core of spirituality: spirituality as a source of meaning or belief which leads to connectedness with something greater than oneself; (2) A function of spirituality: spirituality empowers people to cope in a crisis, by providing motivation, hope and comfort; (3) The experience of spiritual crisis: admission to hospital or residential care can lead to existential struggle; and (4) The spiritual practice: of holding space between struggle and growth. Greater understanding of the theoretical basis of their work may allow chaplains to offer more in the therapeutic space.

## Introduction

It is widely acknowledged that holistic person-centered healthcare encompasses the biological, psychological, social, and spiritual aspects of a person’s well-being (Timmins et al., 2019; World Health Organisation, 2014). Spirituality has been defined as “the dynamic dimension of human life that relates to the way persons (individual and community) experience, express and/or seek meaning, purpose and transcendence, and the way they connect to the moment, to self, to others, to nature, to the significant and/or the sacred” (Nolan et al., 2011).

Research suggests that spirituality has an important role to play when a person enters hospital or an aged care facility, and may be associated with a range of positive health outcomes including coping, quality of life, life satisfaction and lower levels of psychological distress (Amiri et al., 2021; Jones et al., 2018; MacKinlay & Burns, 2017). It may also contribute to negative outcomes such as religious struggle and negative coping methods (Pargament et al., 1998).

A recent Delphi study examining the research evidence for spirituality and health outcomes recommended that: (1) spiritual care should be routinely incorporated into the medical care of patients with serious illness (92.5% consensus); (2) spiritual care education should be included in the training of members of the interdisciplinary medical team (76.9% consensus); and (3) chaplains (also known as spiritual care practitioners and pastoral care providers) should be included in the care of patients with serious illness (69.2% consensus) (Balboni et al., 2022).

In Australia, chaplains deliver dedicated spiritual care across both hospital and aged care settings (Spiritual Care Australia, 2020). Current training requirements and employment arrangements for healthcare chaplains vary across different states in Australia. Spiritual Care Australia, a national association for chaplains, has a minimum standard for certification which includes a degree or diploma in theology, philosophy, religious studies or humanities and at least one 400-h Clinical Pastoral Education unit or an equivalent supervised reflective practices training module (Spiritual Care Australia, 2022).

The Spiritual Health Association has developed a comprehensive capabilities framework for spiritual care practitioners in health care but currently many Australian chaplains would only meet the criteria for the first of four levels (Spiritual Health Association, 2020). Some chaplains are employed directly by the healthcare facility, however, many are recruited and employed by religious organizations and seconded to hospitals as is the case through the Civil Chaplain Association Committee in New South Wales (Civil Chaplains Association Committee, 2010). Chaplains may only visit people of their religion/denomination or provide ward-based spiritual care, but many do both.

Australia is a rapidly changing and culturally diverse nation with a rich and vibrant indigenous history (Isaacs, 2009). National census figures reveal a trend away from formal religion, with the number of Australians identifying with “no religion” increasing from 19% in 2006 to 30% in 2016 and 38% in 2021 (Australian Bureau of Statistics, 2022). There is also increasing religious diversity. Although Christianity is the nation’s most common religion (43.9%), the fastest growing religions are Hinduism (2.7%) and Islam (3.2%) and almost half of Australians have a parent who was born overseas (Australian Bureau of Statistics, 2022). Despite many Australians not considering themselves to be religious, almost half consider themselves spiritual, particularly since the COVID-19 pandemic (McCrindle Research, 2021).

Alongside these changes is a growing acknowledgment that the spirituality of Australian Aboriginal and Torres Strait Islanders may benefit the spiritual well-being of all Australians (Pinkstone, 2021; Ungunmerr, 1988; Yap & Yu, 2016). Exposure to the indigenous experience of spirituality in Australia makes visible valuable concepts such as one’s mob, country, home, and being grounded in space and time, which open up discussions that may be relevant for many people (Tacey, 2009). Recent studies suggest cultural factors may play a significant role in how spirituality is understood, and that obtaining Australian perspectives on this topic is important (Best et al., 2022; Jones et al., 2021).

While several studies have considered the role of Australian chaplains in healthcare (Best et al., 2020a, b; Drummond & Carey, 2020; Jones et al., 2020), few have considered chaplains’ perspective on spirituality itself. Koenig has noted the evolution of definitions of spirituality in the secular context (Koenig, 2008). The aim of this study was to explore Australian chaplains’ perceptions of spirituality and how it helps people at times of crisis. This study data was collected as part of a larger study, which explored the response of Australian health and aged care chaplains during the COVID-19 pandemic (Jones et al., 2020).

## Methods

### Data Collection

Ethical approval for this study was obtained from the University of Notre Dame Australia Human Research Ethics Committee (2020-061S). An email invitation was sent to members of Spiritual Care Australia, a national professional association of chaplains. Only those working as a chaplain in a health or aged care setting were eligible. Informed consent was provided by all participants prior to being interviewed. Participants were purposively sampled to include as broad a range of demographic variables as possible.

Qualitative interviews were conducted by KJ, a researcher with a background in healthcare (not a chaplain), between May and September 2020. Zoom video conferencing software was used to conduct semi-structured interviews, which lasted approximately 45–60 min. All participants were asked about their perspective on human spirituality and invited to discuss its impact on patients or aged care residents during times of crisis. The remainder of the interview questions focused upon the COVID-19 pandemic, and the results are reported elsewhere (Jones et al., 2020). Questions were generated by the researchers, which included a chaplain, in concordance with a review of the academic literature.

Data was collected until thematic saturation (no new information after three consecutive interviews). Interviews were recorded and transcribed verbatim by a professional service.

### Data Analysis

A reflexive thematic analysis according to the six guidelines provided by Braun and Clarke (2006) was used to analyze the data: familiarization with the data; generating initial codes; searching for themes; reviewing themes; defining and naming themes; and producing the report. Familiarization was achieved by reading and rereading the professional transcription of the interviews. Three of the authors (MB, KJ and KB) independently coded the data, using line-by-line analysis, to generate initial codes. The three researchers then met to compare and review these preliminary codes, and to apply them to the rest of the data.

Draft themes were then defined, named and summarized. Differences were resolved through discussion. The different backgrounds of the researchers (medicine, social work and chaplaincy) enriched these discussions, with each member’s perspective and disciplinary knowledge contributing to reflexivity and a deeper understanding of the data.

## Results

Sixteen chaplains were interviewed. Each worked in a health or aged care setting in one of five Australian states (New South Wales, Victoria, Queensland, Tasmania or South Australia). Three-quarters of the participants were aged over 50, more than 60% were female, and all but one described themselves as Christian. All participants strongly agreed with the statement “I am a spiritual person,” with over half agreeing or strongly agreeing with the statement “I am a religious person.” Only one participant identified with an ethnicity other than Australian, and none of the participants identified as Aboriginal Australian (see Table 1).

**Table 1:** Participant demographic characteristics (*N* = 16)

Item	*N* (%)
*Gender*	
Male	6 (37.5)
Female	10 (62.5)
Age group 20–29	0 (0.0)
30–39	1 (6.3)
40–49	3 (18.8)
50–59	9 (56.3)
60 or above	3 (18.8)
Years of work experience: M (SD)	11.5 (8.0)
*Role*	
Spiritual care practitioner	10 (62.5)
Spiritual care manager	6 (37.5)
*Setting*	
Inpatient care	12 (75.0)
Inpatient and community	1 (6.3)
Aged care (residential)	3 (18.8)
*Ethnicity*	
Australian	15 (93.8)
Asian	1 (6.3)
*Religious affiliation*	
Christian	15 (93.8)
Unitarian	1 (6.3)
I am a spiritual person strongly agree	16 (100.0)
Agree	0 (0.0)
Neither agree nor disagree	0 (0.0)
Disagree	0 (0.0)
Strongly disagree	0 (0.0)
*I am a religious person*	
Strongly agree	4 (25.0)
Agree	5 (31.3)
Neither agree nor disagree	5 (31.3)
Disagree	2 (12.5)
Strongly disagree	0 (0.0)
*Spirituality training*	
Certificate/diploma	4 (25.0)
Bachelor or master’s degree	12 (75.0)

### Themes

**Fig. 1:**
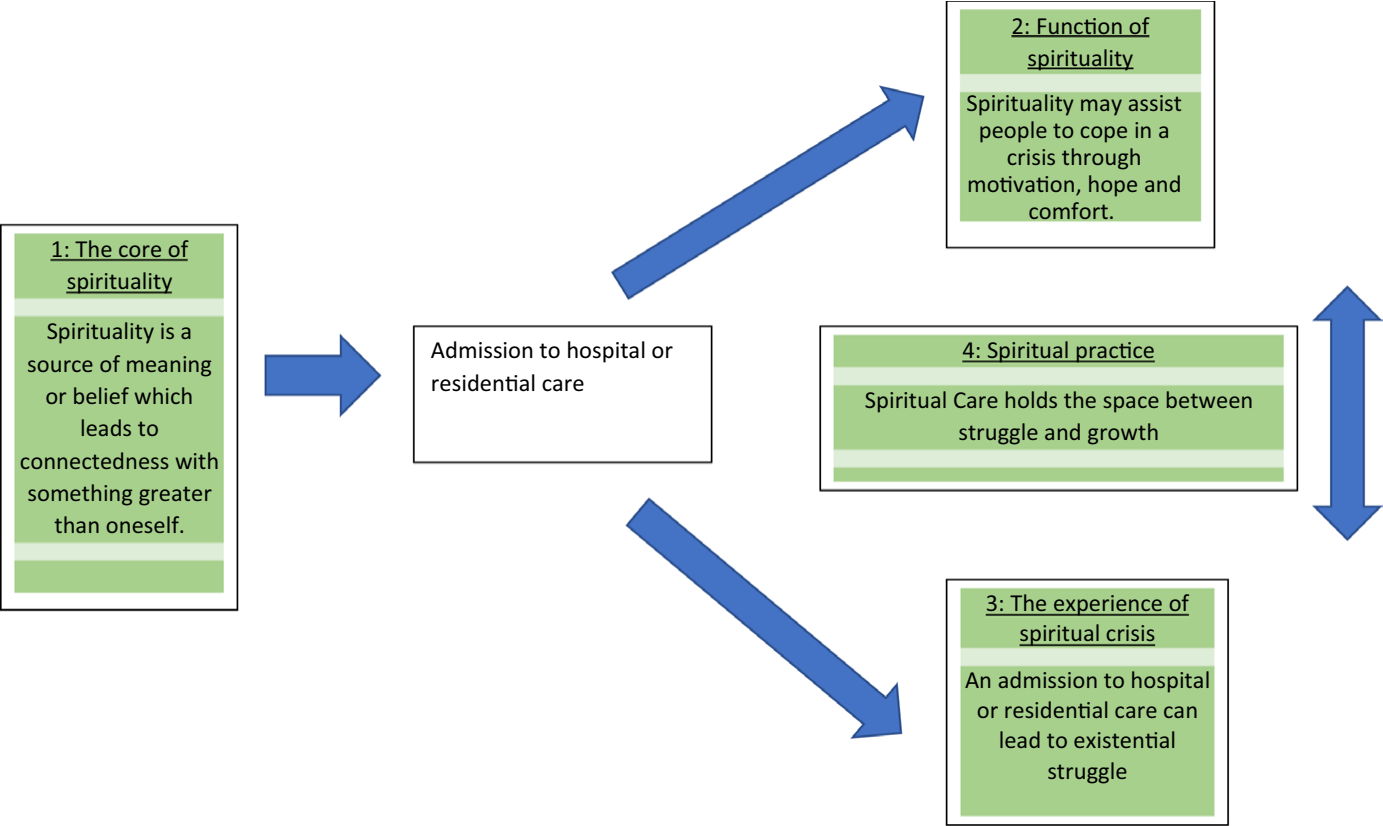
Relationship between study themes

A total of four inter-related themes were identified from the data (see Fig. 1): (1) The core of spirituality: Spirituality as a source of meaning or belief which leads to connectedness with something greater than oneself; (2) A function of spirituality: Spirituality empowers people to cope in a crisis, by providing motivation, hope and comfort; (3) The experience of spiritual crisis: An admission to hospital or residential care can lead to existential struggle; and (4) The spiritual practice of holding space between struggle and growth.

#### Theme 1: The Core of Spirituality

Spirituality at its core was viewed by participants as a source of meaning or belief which connected with something greater than oneself. One participant working in aged care commented how her perspective on sources of spiritual strength had expanded over time to encompass many different sources of meaning beyond religion—sustaining, explaining, and persevering through the ups and downs of life:

It’s what holds you up when life is difficult - what helps you make sense when life is difficult or when life is really good. There would have been a time I think when I thought it would be just faith and people’s faith background or faith tradition, but it’s much broader for me now, you know. I speak to a lot of people who don’t think of themselves as religious at all but there’s a lot of things that are meaningful for them (Participant 6).

The analogy of a tree was used by one participant to describe how sources of meaning and belief could help patients and residents connect with something bigger than themselves—foundational, life sustaining, transcendent, and safe:

“it [spirituality] is the roots of their tree…what they need to get to survive as a human being. To connect them to something greater than themselves, to give them a sense of who they are and something to support them in the vulnerability that they find themselves in” (Participant 1).

Different sources of meaning mentioned by participants included religious faith, the natural world and relationships with other people, all representing connections of different kinds.

Religious beliefs were considered by some participants to be particularly helpful in times of hardship, because most faith traditions include some acknowledgment that suffering will occur. Such beliefs connected the person to the wisdom of ancient traditions. One participant explained:

It gives a world view…with Christianity anyway, I actually expect suffering…I expect that suffering will be part of my life…the Christian patients see that Jesus is with them in that circumstance, and [that] there was a further hope to come (Participant 21).

One participant explained how some religious traditions might help a patient to meditate on suffering in a ritual form and realize an explanatory power through the experience. Such rituals could put “*words around something that is very hard to describe…and speaks to our deeper levels*” (Participant 33). Religious beliefs also helped participants feel less alone, and gave patients connection through a vertical relationship with the transcendent “*that no matter what I go through my God is with me*” (Participant 33).

Participants described how a source of meaning or belief based in the *natural world* could also lead to meaningful connection with something greater than themselves, which gave a sense of safety. Some referred to a connection to a particular place, such as the connectedness to land and country experienced by many Australians, especially Aboriginal Australians. One participant explained how the connection to “country” experienced by Aboriginal Australians was something with which many could identify: “*for some it may be literally the dirt that they stand on, for others it’s the identity and the place that they call home or the place that they feel safe and feel connected with*” (Participant 17). Another participant observed how much the aged care residents she worked with connected to the natural world through just being able to touch elements of the natural world, such as when gardening.

For others, spiritual connectedness was experienced through connection with others, through horizontal relationships such as “*around a dinner table with old friends*” (Participant 10). One participant commented *“when people are struggling, being exposed to an authentic presence of another human being who actually cares about them and for them, that’s the thing that brings spiritual wellbeing”* (Participant 1). Often this connection with others provided a sense of belonging, and patients knowing *“they’re not alone in their struggle, that they have help and comfort from someone outside of themselves”* (Participant 28).

#### Theme 2: A Key Function of Spirituality

Spirituality functions to empower people to cope at times of crisis by providing motivation, perseverance, hope and comfort. Spirituality was understood as a motivating force which sustains and invigorates people, by enabling them to get “*out of bed in the morning*” (Participant 17), “*keeps you fighting*” (Participant 13) and of patients, “*what sets them on fire, what makes their face light up*” (Participant 15). This motivating force helped people to get through “*tough stuff*” (Participant 14) and gave meaning and purpose to their life. Spirituality could also give people a sense of a future, and a hope of something beyond this life.

Alongside hope, spirituality could engender comfort. Placing their situation into a bigger framework or picture helped people to interpret their experiences, “*and find some comfort or hope in that*” (Participant 4). Another participant, working in palliative care, encapsulated much of this when observing, “*spirituality can help calm [patients] and give them the ability to get through [the end of life period], and sustenance and whatever it is that they need to have the strength to get through it”* (Participant 22). One participant described spirituality as a stabilizing influence in times of turmoil or change. She explained: *“it’s like travelling at sea… the ship has to be very anchored otherwise we would be tipped upside down in that sea storm”* (Participant 9)*.*

#### Theme 3: The Experience of Spiritual Crisis

For many people, change, such as that which occurs when entering a health or aged care facility, is often experienced as a point of crisis or a potential turning point for the individual. This crisis may involve a range of different circumstances, which include unexpected injury or illness, loss of independence, or the need for end of life care. According to the participants in this study, such crises can lead to existential struggle, potentially resulting in spiritual distress, but it can also lead to a point of differentiation and choice that leads to spiritual growth, or, in some cases, both distress and growth at different parts of the trajectory. Participants explained that “*in a crisis, that’s when meaning-making spirituality actually comes to the forefront*” (Participant 25): the “*existential angst*” leads the sufferer to place events within a deeper framework or view them against a farther horizon of significance.

One participant working in aged care described the paradoxical nature of the relationship between spiritual distress and spiritual growth: *“it feels painful and very hard, but yet, there’s growth at the same time…there’s a paradox there where you find yourself searching more deeper for greater meaning”* (Participant 2)*.* He went on to say how in aged care this paradox was particularly associated with fear of death and dying. Growth occurred when residents were able to enter the reality of the present and face their fears which led them to view life (and death) differently. Such spiritual growth may be facilitated when aged care residents are provided with an opportunity to explore their fears in a safe and trusting environment, as explained below.

The idea of choosing how to respond to a crisis was also raised by a participant working in a hospital setting. According to this participant, three choices faced a patient with spiritual beliefs at a time of crisis. They might “*lose their spirituality and toss it out the window, hold blindly to their spirituality and not allow themselves to be challenged by the questions, or be open…sit with the tension* [they] *feel between* [their] *experience and* [their] *spirituality*” (Participant 33). The participant went on to say how grappling with suffering was “*what a lot of spirituality is about in times of crisis…being able to hold that tension between what you believe and the questions that the suffering actually presents.*”

#### Theme 4: The Spiritual Practice of Holding Space

The fourth theme identified a spiritual practice enacted by the chaplains whereby they held a “safe space,” which allowed a person to explore and grapple with the spiritual questions posed by their situation. Participants recalled times when a patient was experiencing deep suffering, for whom they were aware that they did not have any answers or words to give. One said “*I sometimes say to patients…I wish I could take this from you and I know I can’t. But I’m here*” (Participant 33). He went on to use the phrase “*holding the space*” to describe his actions and explained that this was about “*naming what is and not judging it.*”

The ability to stay with patients experiencing deep suffering could co-exist with a sense of helplessness for the chaplain. Another participant, who primarily cared for Christian patients who were experiencing distress in the end of life context, recalled one encounter:

When I visited her…she’s exhausted, she’s physically spent, she’s in mental anguish and just at that point there didn’t seem to be any help or hope I could give her...for that person there just didn’t seem to be any hope at that time (Participant 28).

He went on to describe how this young patient had a strong Christian faith to which he could relate:

When I was younger I went through something like this myself…perhaps it’s just [that] your emotions and your distress and sadness can overwhelm your faith for a little while, you know, dark night of the soul…until something brings you out of that…your spirituality or faith would bring you out of that, comfort you, perhaps lift you up a little bit higher. But there are times where you are just right down at the bottom…even the strongest person can feel like that (Participant 28).

To be able to “hold the space” chaplains needed to be able to listen to stories. In one aged care facility the chaplain held a knitting group for residents to facilitate spiritual care. She spoke of how fascinated she was by their stories, “*every day there will be something else I know about them*” (Participant 10). In another example, a chaplain at a children’s hospital built relationships and listened to stories by playing card games with the patients and spending time on the ward with their families. Several participants highlighted the value of just sitting by a patient’s bedside “*and they’ll tell you a story*” (Participant 28). It was important that the topic of the conversation was chosen by the person themselves:

If they want a prayer, we do a prayer, if they want to talk about who’s going to win the footy on the weekend, we talk about that. If they want to talk about how difficult it is accepting going through the dying process, we talk about that (Participant 33).

Chaplains explained that this work was a skilled practice, and that listening to stories was more than just practicing presence and accompaniment, incorporating the utilization of therapeutic counseling skills.

Other chaplains who were engaged in narrative work found working with patients and residents’ stories an opening to the deeper questions of life. One participant described this as *“the bread and butter of spiritual care”* (Participant 1). Another reflected how patients with a Christian faith who are facing death might ask questions such as “*Am I okay?”,* “*Does God still love me?”* (Participant 9). She understood her role to be there to reassure them of God’s love.

A participant working in aged care spoke of the beauty and poignancy in moments of sadness and bearing witness to the perseverance of patients, “*I see lots of moments of real courage …I couldn’t really explain that to anyone else outside here, but there’s something really special about it”* (Participant 10). She reflected that in her previous work as a nurse she had not had the time to notice these poignant moments. Now as a chaplain, she described her role as having *“presence, day in, day out, of being there…and then when the time comes for someone, when they’re dying, they trust you enough to sit with them”* (Participant 10).

## Discussion

The aim of this study was to explore Australian chaplains’ perceptions of spirituality and how it helps people at times of crisis. The 16 participants in this study held a comprehensive view of spirituality, which extended beyond religion and encompassed meaningful connectedness: to others; to nature; to God or something beyond oneself. The participants describe spirituality in line with Nolan et al.’s working definition of spirituality which highlights the dynamic dimensions of life and the way in which people experience their reality, express it and seek for deeper meanings and possibilities. For many people these connections have a transcendent quality across a range of relationships with time, the self, other people, nature or that which is “other” (Nolan et al., 2011).

It is notable that the participants preferred to use metaphors and analogies rather than defining spirituality either over and against religion, physical health, psychology and social work, or by defining and explaining the boundaries and overlaps between the adjacent disciplines. Metaphorical language allows the participants to say what spirituality *is*, rather than being limited to what it *is not*. However, despite lack of technical definitions, or indeed perhaps because of it, the participants demonstrate a robust concept of spirituality touching four interdependent aspects of spirituality as described in the consensus statement above, or the “four C’s” of religion: creed (propositional beliefs), code (codes of practice), cult (rituals) and community (Albanese, 2012) or (alternatively) meaning, purpose, community and rituals (Burton, 2020). This can be summarized as: meaning/beliefs/values (built up over time); purpose/possibilities/hope (to do with the future); connections/belonging/relationships (current relational web); and transcendence/sacred/divine (vertical or other dimension to life).

The first theme described spirituality at its core providing a source of meaning or belief which leads to connectedness with something beyond oneself. Metaphorical imagery such as the roots of a tree, the anchor of a ship, and a connection to people or land, were used to illustrate a tethering and locative nature of such meaningful connections. Although international conceptualizations of spirituality make mention of the importance of connection to the natural world (Davis et al., 2015; de Jager Meezenbroek et al., 2012; Naor & Mayseless, 2020; Puchalski et al., 2014), a profound and unique spiritual relationship exists between Aboriginal and Torres Strait Islanders, their people or “mob,” and their homeland or “country.”

For Aboriginal Australians, spirituality encompasses belonging and identity, giving ‘meaning to all aspects of life including relationships with one another and the environment’ (Grant, 2004, pp 8–9). At the end of life, the relational aspect of spirituality is emphasized for Aboriginal Australians, who often express a deep longing to be with their people and in their physical homeland at the point of death (Palliative Care Australia, 2021; Pinkstone, 2021). Many aspects of indigenous spirituality are being embraced in Australia, which may influence how spirituality is perceived in health and aged care for all Australians, as well as in the wider community (Ungunmerr, 1988; Yap & Yu, 2016).

Metaphorical language is central to ideas of Aboriginal spirituality. Examples include: “spirituality is oneness,” “the land is my mother,” or “the land is my backbone.” In his book *The Rule of Metaphor*, French philosopher Paul Ricoeur explores live metaphors, which are short sentences that work with the pre-existing stock of language (Ricoeur, 2003, p 186) but introduce a “twist” that creates new meaning by placing two familiar ideas alongside each other (op. cit., pp 114, 272, 344). Live metaphors contrast with dead metaphors such as the face of a clock, or leg of the chair, where an expression has become a cliché. Living metaphors are sentences that do not make literal sense.

Metaphorical sense emerges through an intentional category mistake when two words or ideas from different semantic ranges are employed, without violating the sematic boundaries of either word, but the subject and predicate are allowed to reverberate against each other in a creative tension. The tension pushes the reader or hearer into a new metaphorical space that allows us to say more than either of the two words alone could convey, and creating cognitive disruptions or as one commentator phrased it, “forcing the language to jump fixed tracks” (Kort, 1979). The metaphor establishes another world that corresponds to new possibilities which may include inbreaking of “the other,” giving words in spiritual discourse for which there are none. Ricoeur’s test for the validly of a metaphor is illumination: does this particular metaphor make my experience of the world more meaningful or intelligible? (Vanhoozer, 1990, p 67).

The validity of the reported metaphors can be tested by placing them in their broader context, as has been done by Indigenous Australians. Playwright Mudrooroo extends the idea of “spirituality as oneness” to include “interconnectedness with all that lives and breathes, even with all that does not live or breathe” (Mudrooroo, 1995, p 33). Uniting Church pastor, Djinyini Gondarra, explains “This land is my mother. Like a human mother the land gives us protection, enjoyment, and provides for our needs—economic, social and religious. We have a human relationship with the land: mother—daughter/son*”* (Gondarra, 1985). Musician Galarrwuy Yunipingu says, “The land is my backbone… I only stand straight, happy, proud, and not ashamed about my colour because I still have land… I think of land as the history of my nation” (Calma, 2008).

In each case the context around the metaphor enhances it, which in turn adds new layers to the metaphorical meaning, a process Ricoeur called a hermeneutical circle. When the metaphor and context are taken together, rather than the removal of conflict, a process of mental mediation and negotiation begins (Ricoeur, 2003).

By briefly reviewing several metaphors related to Aboriginal concepts of spirituality and the land, it becomes clearer that metaphors help illuminate the inextricably woven vertical and horizontal concepts of meaning, belonging, relationships and time, which hints at the question as to whether this may not be the case for human spirituality more generally. The participants’ responses, when layered together, describe a spirituality in terms of a sustaining life force with an interconnected web of horizontal human connections and vertical connections with transcendent realities. Spirituality as described was seen as foundational with interpretive and explanatory power through life’s “ups and downs” connecting into ancient wisdom traditions. This is consistent with that found in other populations (Mercadante, 2014). None of the participants viewed spiritual “meaning” as an end in itself but saw it very closely associated with connection, relationship and belonging, confirming previous observations in the end of life context (Mount et al., 2007).

The second theme described a function of spirituality, to empower people to cope in a crisis, providing motivation, hope and comfort. Across the data it can be observed that spirituality of patients and residents was experienced as invigorating, comforting and stabilizing, supplying perseverance and enabling the recipient of care to move forward into new possibilities. These benefits of spirituality have been previously identified in Australian literature (Spiritual Health Victoria, 2016).

The aspect of empowerment is perhaps most clearly demonstrated in this data set by chaplains of a faith concordant with the patients or residents. In several examples, the chaplain could identify with some of the questions with which the person was grappling and used their familiarity with the common belief system to speak comfort into the situation. It seems there may be additional comfort and solace that can be provided by chaplains who share one’s faith. When appropriately done, to ensure the beliefs of the chaplain are not projected on to the patient, this can be encouraged or supported in health and aged care settings without reduction in satisfaction with care (Liefbroer & Nagel, 2021).

The third theme described the existential struggle experienced at times of change or crisis. The previous theme is only part of the spiritual care picture as many others experience significant spiritual distress rather than empowerment at times of crisis, change or chronic shortfall of health or needs. This is a well-known consequence of the experience of loss (Best et al., 2015). Such people are described as facing the seeming unfairness of a situation which can cause them to feel as if they are falling backwards or have had the rug pulled out from under their feet.

The participants in this study describe people being faced with a three-way choice in the face of unfortunate events between giving up, grinning and bearing it, or entering into the paradoxical mystery of events. The key here is the possibility of differentiation and choice even in these events, but this will depend to some extent on the knowledge, skills and practice of the chaplain to offer a deeper spiritual framework (Gall et al., 2005) or farther horizon of significance (Taylor, 1992) to find a path through the paradoxical nature of facing fears and overcoming them (Cole & Pargament, 1999). This leads directly into the fourth theme of holding the space between struggle and growth.

The fourth theme was exemplified through narrative, another theme developed by Ricoeur. Both metaphor and narrative are creative discourses which employ semantic innovations and work together as a pair across space and time (Vanhoozer, 1990). Ricoeur employs a spatial frame to describe a metaphor whereby two distant meanings are brought together within a proximal space, allowing a transfer of meaning (Ricoeur, 1978). Metaphors carry ideas across conventional boundaries between the known and unknown, or visible and invisible, allowing for a time disparate world to permeate each other. In contrast to metaphors, narratives create a temporal unity between a diversity of character, event, goals and causes (Vanhoozer, 1990).

Polkinghorne proposed that human beings exist in three realms, the material, organic and the realm of meaning (Polkinghorne, 1988, pp 1–10; 157). The organic and material realms are accessed using formal logic (p 3), whereas the realm of meaning is accessed through narrative logic (p 36). Polkinghorne, building on Ricoeur, contended that the narrative form shapes a person's experience of time (p 135), the narrative following linguistic structures that give meaning to presenting realities (p 136).

Both metaphors and narratives redescribe the world and place a new interpretive grid over reality. Storytelling emerged as an important element at times when chaplains “hold the space” between struggle and growth without judgment. As reported by participants in this study, chaplains are in a unique position to hear these narratives or stories, whether it be at the bedside, playing games with a child, or in a knitting group with aged care residents. Cooper (2018) has described chaplains as “story-catchers.” She describes how the telling of a story and hearing it, can be “empowering and emancipating” for both the teller and listener.

Chaplain participants in an Australian study by Best et al., (2020a) noted that they had more time to listen to patients’ stories than other staff, and so were able to build a relationship with patients, thereby opening the conversation to deeper topics. It can be argued that such therapeutic listening provides a way for chaplains to build relationships and “hold the space” which creates opportunities for exploring existential questions.

Related to the theme of stories was the observation by participants that struggle and suffering could, in some cases, lead to growth. The relationship between suffering and post-traumatic growth has been explored by many theorists and practitioners over the years (Frank, 1995; Pargament & Exline, 2022; Richardson, 2002; Tedeschi & Calhoun, 2004).

The concept of growth through suffering has been explored in the book *The Wounded Storyteller* (Frank, 1995). Frank introduces the “chaos” narrative, the “restitution” narrative and the “quest” narrative. Whereas the “restitution” narrative depicts the hope of the person with an illness or injury that life will return back to normal, the “chaos” narrative is where all hope is lost, leaving the person in despair. The “quest” narrative, in contrast, is adopted by the person who “meets suffering head on” and seeks to use it, embarking upon a journey of growth and discovery.

The concept of growth depicted in the quest narrative is reinforced by Pargament and Exline (2022) who have suggested that growth may not only “refer to resilience and recovery of health and mental health after a spiritual struggle but also to positive, potentially transformational changes in the individual’s larger purpose in life and orientation to him or herself, the world and the sacred” (p 85).

The data demonstrates that the chaplains who participated in this study demonstrate an intuitive understanding of the power of compassionate presence and spiritual hospitality. The evidence demonstrates tenets of trauma-informed care: namely safety, trust, choice, collaboration, empowerment and respect for values (Center for Preparedness & Response, 2020; NSW Health, 2022). The practices of presence and hospitality focus on attention, attunement, and accompaniment. When the participants bear witness to the suffering of others, giving words to suffering and distress without judgment, they are endeavoring to avoid drama triangles, not aiming to fix anything but holding space to allow for their work and/or that of God and others to work. They are skilled at drawing on narrative work and the deep accumulated wisdom of religious and philosophical systems.

The participants for the most part accessed their descriptions of spirituality focusing on the patients’ stories, reflecting both their own and the patient perspective, suggesting that they adopted an experiential (rather than theoretical) understanding of spirituality. None of the participants made reference to conceptual models of spirituality and spiritual care, such as those outlined by Pargament and Exline (2022). Perhaps chaplains could offer more in the therapeutic space if the theoretical basis of their work was deepened through greater emphasis in their training, particularly in areas of ontology and epistemology. Avenues of training that would be enhance chaplains’ work with metaphors and narratives could include training in Clean Language technique (Wilson, 2017) or Narrative Therapy/Practice (White & Epston, 1990). Such training would help the chaplains become more conscious of the inner workings of the metaphors and narrative with which they are intuitively engaging.

The research participants demonstrated broad rather than narrow concepts of spirituality which attend to all of life, rather than just pertaining strictly to religious matters. Moreover, even when religious matters are addressed, they are viewed within an all-of-life framework. A second but no less insignificant point is that the spiritual care here described is not only the purview of the specialist spiritual care practitioner but also the generalist, and that it and compassionate presence are the responsibility of all health care staff, as outlined in the generalist/specialist spiritual care model used in many palliative care units (Balboni et al., 2014). In this palliative model a patient’s spiritual care is incorporated into all aspects of a patient’s care with patients experiencing spiritual distress and unmet spiritual needs being referred to a chaplain (Best et al, 2020b).

This contrasts with the misconception that spiritual care is an isolated addendum to care concerned only with religion, a misunderstanding exemplified in a recent cross country analysis of the quality of death and dying (Finkelstein et al., 2022). However, even Finkelstein and colleagues found evidence that good end of life care was characterized by factors identified in the four themes reported by our participants: attention to core spiritual beliefs, the role of spirituality in helping people cope with their situation, trauma-informed care to manage the spiritual crisis which may be precipitated by a serious medical diagnosis, and the need for the spiritual practices that provide literal and metaphorical safe places to allow for people to grapple with their new reality and its deepest meanings.

### Limitations

Limitations of this study include a lack of religious or ethnic diversity among participants, with the majority of participants identifying as Christian and only one participant identifying with an ethnicity other than Australian, although this does reflect the current workforce. None of the participants identified as Aboriginal Australian. Furthermore, we only collected views of chaplains, and perspectives of other staff members, patients or residents may differ. All interviews were conducted during the COVID-19 pandemic, so may have captured perspectives that were influenced by the unique circumstances of the period.

### Conclusion

The findings of this study show that chaplains in Australia have adopted broad conceptualizations of spirituality, linking it with life, meaning and connectedness. Participants employed metaphor and narrative to describe spirituality. In the context of hospital or residential aged care admission, spirituality was perceived to be something that a person could draw upon to cope with their distress. Spiritual care is associated with accompanying patients and aged care residents during times of distress, suffering and isolation. Key to this support is creation of a safe space, understanding the importance of spiritual struggle and spiritual growth, strong relationships where stories are heard, and the ability of the chaplain to stay with the person even when they may feel helpless.

Chaplains may benefit from greater emphasis in their training on the theoretical basis for their work. This may assist them in their understanding of the inner workings of the metaphors and narrative with which they are intuitively engaging, thereby helping them to offer more in the therapeutic space.
